# Semen Quality in Males Suffering From COVID-19: A Pilot Study

**DOI:** 10.7759/cureus.31776

**Published:** 2022-11-22

**Authors:** Satish P Dipankar, Tribhuwan Kumar, Afreen Begum H Itagi, Bijaya N Naik, Yogesh Kumar, Mona Sharma, Asim Sarfaraz, Amita Kumari

**Affiliations:** 1 Physiology, All India Institute of Medical Sciences, Managalagiri, IND; 2 Physiology, All India Institute of Medical Sciences, Patna, IND; 3 Physiology, All India Institute of Medical Sciences, Mangalagiri, IND; 4 Community and Family Medicine, All India Institute of Medical Sciences, Patna, IND; 5 Physiology, India Institute of Medical Sciences, Patna, IND; 6 Reproductive Biology, All India Institute of Medical Sciences, New Delhi, IND; 7 Microbiology, All India Institute of Medical Sciences, Patna, IND

**Keywords:** spermatogenesis, sars-cov-2, male infertility, dna fragmentation, semen, covid-19

## Abstract

Background

It is well known that some viral infections may affect male fertility. Coronavirus disease (COVID-19) can lead to multiorgan damage through the angiotensin-converting enzyme-2 receptor, abundant in testicular tissue. However, little information is available regarding the shedding of severe acute respiratory syndrome coronavirus-2 (SARS-CoV-2) in semen and its impact on spermatogenesis and fertility potential. We planned to investigate the presence of SARS-CoV-2 in the semen of COVID-19 males and to study the effect of COVID-19 on semen quality and sperm DNA fragmentation index.

Material and method

Thirty COVID-19 male patients aged 19-45 registered to AIIMS Patna hospital participated in the survey between October 2020 and April 2021. We conducted a real-time reverse transcriptase test on all the semen samples. Detailed semen analysis, including the sperm DNA Fragmentation Index, was done at first sampling that is during COVID-19. After 74 days of the first sampling, we obtained the second sampling and repeated all the above tests.

Results

All semen samples collected in the first and second sampling tested with real-time reverse transcription-polymerase chain reaction (RT-PCR) were negative for SARS-CoV-2. In the first sampling, semen volume, vitality, total motility, sperm concentration, total sperm count, % normal morphology, % cytoplasmic droplet, and fructose were significantly lower. In contrast, semen agglutination, % head defect, DNA Fragmentation Index, liquefaction time, semen viscosity, and leukocytes were increased. These findings were reversed at the second sampling but not to the optimum level. All these findings were statistically significant (p < 0.05 for all). Thus, COVID-19 negatively affects semen parameters, including sperm DNA fragmentation index.

Conclusion

Although we could not find SARS-CoV-2 in the semen, the semen quality remained poor until the second sampling. Assisted reproductive technology (ART) clinics and sperm banking facilities should consider assessing the semen of COVID-19 males and exclude men with a positive history of SARS-CoV-2 until their semen quality returns to normal.

## Introduction

Since the severe acute respiratory syndrome coronavirus-2 (SARS-CoV-2) outbreak in December 2019, coronavirus disease (COVID-19) has declared a global pandemic on March 11, 2020 [1]. The spread of SARS-CoV-2 from person to person through respiratory droplets is well-established [2]. A recent study reported SARS-CoV-2 in stool, saliva, and urine samples, suggesting other means of transmission [3]. Kevadiya et al. supported the presence of SARS-CoV-2 in several human secretions like throat, saliva, pulmonary alveolar washing, and feces [4]. Another study stated that shedding of the coronavirus is possible but extremely rare [5]. There is evidence of the relationship between members of the coronavirus family and orchitis in humans [6] and cats [7]. Severe acute respiratory syndrome coronavirus (SARS-CoV) is known to damage multiple organs, including the human testis leading to widespread germ cell destruction in the seminiferous tubules. SARS-CoV-2 also has angiotensin-converting enzyme-2 (ACE2) as one of the significant receptors that mediate the entry of SARS-CoV-2 into human cells. ACE2 receptors are abundantly present in the testis and male reproductive tract [8]. Men are more susceptible to COVID-19 than women because of the high expression of coronavirus receptors, ACE2, and lifestyle, such as the higher practice of smoking and alcoholism among men. Therefore, the death rate of COVID-19 among men is 4.7% compared with 2.8% for women [9]. Thus, men are more vulnerable to COVID-19 including its effect on the male reproductive system.

Objective

The objective is to detect the presence of SARC-CoV-2 in semen and its effect on semen quality thereby providing insights into the early impact on male reproductive function.

## Materials and methods

Study sample size

Due to many limitations during the first wave of COVID-19, we conducted this pilot study over six months. The sample size of the prospective longitudinal cohort study was 30.

Study Participants

We included COVID-19-positive males diagnosed at the All India Institute of Medical Sciences (AIIMS), Patna, a dedicated COVID-19 hospital. After IRB approval (Institutional Ethics committee, AIIMS Patna, RC/AIIMS/Pat/2020/53), study participants were isolated from October 2020 to April 2021; their ages ranged from 19-45 years. Laboratory confirmation for COVID-19 was done based on the WHO guidelines [10]. We telephonically contacted study participants and received informed verbal consent.

Inclusion criteria

Mild cases of COVID-19 men, not suffering from dyspnea, married males having at least one child, and abstinence period of two to seven days.

Exclusion criteria

History of infertility, severe COVID-19 disease, negative real-time reverse transcription-polymerase chain reaction (RT-PCR) test of SARS-CoV-2 of their nasopharyngeal swab sample, sexually transmitted diseases based on the history and earlier investigations, history of immunosuppression, history of smoking, alcohol intake, diabetes, varicocele.

Study tools

Safety Measures

Semen collection and handling of the semen samples were done following all biosafety precautions.

History of Participants

We collected the history of COVID-19 progress and we identified sexually active males willing to participate in the study. We also assessed COVID-19 history and reproductive quality of life with the sex partner using the FERTIQOL (Fertility Quality of Life) questionnaire along with routine history taking in our lab. Participants with an adequate score of > 70 continued the study [11]. Other clinical history and information about their investigations like complete blood count, blood biomarkers, and chest x-ray findings were collected.

Basic Semen Analysis

Semen samples of participants were collected by masturbation in a sterile container maintaining a two to seven days abstinence period. Immediately after the liquefaction of semen at room temperature, semen analysis was done in the andrology laboratory according to the WHO laboratory manual for the 'Examination and Processing of Human Semen' 5th edition 2010 [12].

Seliwanoff's Test for Seminal Fructose Estimation

Fructose is qualitatively assessed for presence or absence in seminal plasma using a simple color change test. Seliwanoff’s reagent (resorcinol in hydrochloric acid) was utilized for this test. A reasonable amount of seminal fructose develops a cherry red color after adding semen with Seliwanoff’s reagent in a test tube kept in a hot water bath [13]. Fructose is the energy source for spermatozoa and acts as a marker of seminal vesicle functionality that may be altered during viral infection [14].

Testsimplets Test for Leukocytopenia

A readymade glass slide coated with stain containing methylene blue-N and cresyl violet acetate was used. A drop of the semen ejaculate is placed in the center of a microscope slide coated with a staining medium, a cover glass is then applied to the microscope slide and the preliminary microscopic inspection carried out after only 15 min. Leukocytospermia is considered to exist if more than one million leukocytes per milliliter are detectable in the ejaculate or if, during microscopic differentiation at 400x magnification, more than four leukocytes are detectable in each high-power field (HPF) [15].

Sperm Vitality

On a glass slide a drop of semen was taken, add two drops of 1% aqueous Eosin Y. Over it, mix well, and wait for 15 seconds. To this mixture, add two drops of 10 % aqueous Nigrosin. Again, mix well and take 10µL drops of the mixture on the new glass slide. Make a thin uniform smear and allow it to air dry. Observe under oil emersion objective lens and count the white and pink sperms. Live sperms remain unstained and look white while dead sperms will get stained due to the loss of their cell membrane integrity. Count a total of 200 sperms and estimate the percentage of white sperms. Normal sperm vitality is more than 58% [16], the same is discussed in the WHO manual of semen analysis [17].

DNA Fragmentation Index (DFI)

*A*ssessed with a kit-based sperm chromatin dispersion test using Qwik Check DFI kits manufactured by Medical Electronic Systems India Private Limited, Chennai, Tamil Nadu, India.

RT-PCR of Semen

To detect SARS-CoV-2 in the semen, after measuring semen volume, a part of the semen sample was transported in viral transport media to the microbiology laboratory of our institute for the detection of viral RNA through RT-PCR. We stored the seminal samples at −20°C and performed Qualitative RT-PCR using a one-step real-time PCR reaction mixture and primer-probe mixture approved by the Indian Council of Medical Research, Government of India. We used the COVID-19 RT-PCR kit prepared by AB Diagnopath Mfg. Pvt. Ltd. Delhi, India. We processed all the semen samples along with positive control and a no template control for validation. We conducted RT-PCR by using real-time either Biorad CFX96 or ABI Quant studio 5 Dx machine with reaction protocol set as per instruction in the kit insert. The nucleic acid extraction is suitable for sperm using the Pure MagNA technique. Only semen samples from patients detected positive for SARS-CoV-2 by RT-PCR on nasopharyngeal swabs were analyzed. We repeated all the above semen tests after 74 days of the negative nasopharyngeal RT-PCR results. In both acute and post-COVID-19 states, semen samples were negative for SARS-CoV-2.

Statistical analysis

The data collected was entered in Microsoft Excel and analyzed using SPSS (Statistical Package for the Social Sciences) version 20.0. We applied Paired “t-test” for all normally distributed variables, marked as the single asterisk (*), and expressed as mean and standard deviation. Wilcoxon signed-rank test was used for all not normally distributed variables, marked as the double-asterisk (**), and were defined as median and interquartile range p-value less than 0.05 was statistically significant.

## Results

High-grade fever was present in all study participants (30/30). Other common symptoms were cough (23/30), dyspnea (00/30), headache (21/30), muscle pain (20/30), anosmia, loss of taste (19/30), and common cold (24/30), weakness, and weight loss (27/30). We conducted complete andrological clinical examinations of all the study participants after their recovery from COVID-19 and before their second semen examination. All study participants were fit; therefore, continued in the research study. Table 1 shows the demographic characteristics of the study participants and the treatments received.

**Table 1:** Distribution of the demographic data of the participants during COVID-19 (n=30)

Characteristics	Values mean (range)
Age range (years)	28.31 (19 – 43)
BMI range (kg/m^2^)	22.13 (18 – 24)
Number of days of symptoms	5 (3 – 7)
Number of participants home quarantined	30
Semen RT-PCR	Negative in all participants
Medical treatment for COVID−19	Doxycycline, Azithromycin, Paracetamol, Montelukast, Multivitamins, and vitamin C

During the first semen sampling, semen quality got affected as follows: Semen volume was below 1.5 mL among 10 study participants. Semen viscosity in 26 participants was more than 02 cm. Liquefaction time among 14 participants was prolonged. Among the 30 participants, sperm agglutination was from grade I to IV. Sperm vitality in 29 participants was below 58%. Sperm motility (progressive and total) in 22 participants was below the average value. Among the 14 participants, the sperm concentration was below 15 million/mL. The total sperm count in 12 participants was below 39 million/ejaculate. In 21 participants, sperm morphology was below 4%. The number of leukocytes in all 30 participants was above 04/HPF, and DFI among all 30 participants was more than 30%. Table 2 shows the impact of COVID-19 on semen parameters.

**Table 2:** Number of participants with their semen parameters outside the reference values as per WHO manual (2010)

Semen parameters (Reference value as per WHO 2010)	Number of participants with their semen parameters affected during first sampling (COVID-19) (n=30)	Number of participants with their semen parameters affected during second sampling (74 days post-COVID-19) (n=30)
Volume < 1.5 mL/ejaculate	10	02
Viscosity > 2 cm	26	21
Liquefaction time > 60 min	14	10
Agglutination > grade 4	30	29
Vitality < 58 %	29	26
Progressive motility < 32 %	22	10
Total motility < 40 %	22	10
Sperm concentration < 15 million/ml	14	05
Total sperm count < 39 million/ ejaculate	12	03
Normal morphology < 4 %	21	05
Leucocytes > 4 cells/HPF	30	23
DFI > 30 %	30	27

During the second semen sampling, semen quality got affected as follows: Semen volume was below 1.5 mL among two participants. Semen viscosity in 21 participants was more than 2 cm. Liquefaction time among 10 participants was prolonged. Among the 29 participants, sperm agglutination was from grade I to IV. Sperm vitality in 26 participants was below 58%. Sperm motility (progressive and total) in 10 participants was below the average value. Among the five participants, sperm concentration was below 15 million/mL. The total sperm count in 03 participants was below 39 million/ejaculate. In five participants, sperm morphology was below 4%. Leukocytes in 23 participants were above 04/HPF, and DFI among all 27 participants was more than 30%. Table 3 shows the comparison of semen parameters during COVID-19 and 74 days post-COVID-19 period.

**Table 3:** Comparison of semen parameters during COVID-19 and 74 days post-COVID-19 period (n=30)

Semen parameter (Normal values)	First sampling (COVID-19)	Second sampling (Post-COVID-19)	p-value
	Mean (SD)	Mean (SD)	
Volume (> 1.5 ml/ejaculate)*	2.25 (0.796)	2.92 (0.678)	0.001
Viscosity (1 – 2 cm)**	4.5 (3 – 20)	3 (2 – 5.25)	0.003
Liquefaction time ( 20 – 60 min)**	40 (33.75 – 60)	30 (24.5 – 40)	0.011
Agglutination (grades 1 -4)*	3.4 (0.6215)	2.833 (0.8743)	0.003
pH (7.2 – 7.8)*	8.187 (0.3589)	8.1 (0.4433)	0.36
Vitality (> 58%)*	33.867 (14.81)	43.933(14.10)	0.014
Progressive motility (≥ 32 %)*	20.2 (09.05)	31.03 (10.46)	0.002
Total motility (≥ 40 %)*	26.4 (11.07)	38.43 (13.48)	0.002
Sperm count (> 15 millions/ml)**	26.85 (13.05 – 39.7)	58.75 (41 – 74.07)	0.000
Total sperm count (> 39 millions/ejaculate)**	46.45 (29.72 – 82.27)	161.6 (100-62 – 205.57)	0.000
Normal morphology (> 4 %)**	5 (3 – 10)	10 (7.75 – 15.25)	0.034
Head defect (%)*	43.6 (8.01)	38.23 (6.9)	0.008
Neck defect (%)*	15 (5.45)	16.93 (6.75)	0.194
Tail defect (%)*	26.17 (5.99)	23.93 (8.04)	0.251
Cytoplasmic droplet (%)**	6 (5 – 8.25)	9 (5.75 – 12)	0.024
WBC (< 4/HPF)**	9 (6 – 13.5)	6.5 (4.75 – 9)	0.002
Fructose (present)*	2.37 (0.49)	2.97 (0.76)	0.002
DFI (<30 %)*	74.25 (8.93)	66.94 (11.98)	0.000
*Normally distributed variables presented as mean (standard deviation), paired ‘t-test was applied **Not normally distributed variables presented as median (interquartile range), Wilcoxon signed-rank test was applied p-value < 0.05 was considered statistically significant, bold values in table 2. indicate significant p-values.			

During the first semen sampling, values of semen volume, sperm vitality, total motility, sperm concentration, total sperm count, % normal morphology, % cytoplasmic droplet, and semen fructose were below their normal values. The values of all these semen parameters increased during the second semen sampling. The increase in these semen parameters was statistically significant (p < 0.05 for all). During the second sampling, values of semen agglutination, % head defect, DFI, semen liquefaction time, semen viscosity, and leukocytes in the semen were below their normal limits. The decrease in these semen parameters was statistically significant (p < 0.05 for all). During both the samplings, pH, % neck defect, and % tail defect was not statistically significant.

## Discussion

Many researchers like Jin et al. showed that although men and women have the exact prevalence of COVID-19, overall, men's health is at high risk, leading to worsened outcomes and death [8]. The sex's different lifestyles, such as smoking addiction, which is more prevalent in men than women, are considered a potential risk factor for developing COVID-19. More estrogen in females has a protective role, and the presence of “XX” chromosomes in women provides more innate immunity than in men.

ACE2 functions as an enzyme as well as a functional receptor on the cell surface through which SARS-CoV-2 enters the host cells; ACE2 is highly expressed in the heart, kidneys, and lungs and is shed into the plasma [18]. The expression of ACE2 is enriched in the renal tubular cells, Sertoli cells, Leydig cells, and cells in seminiferous ducts in the testis [9]. The testis is also one of the organs with high levels of constitutive expression of ACE2, and it raises a concern that the SARS‐CoV‐2 has a route to enter testicular cells and thus could cause damage [19]. Moreover, TMPRSS2 (Transmembrane protease, serine 2) expression, similar to ACE2, was also enriched in spermatogonia and spermatids. Co-expression of ACE2 and TMPRSS2 genes have been detected by single-cell RNA-sequencing analyses on goblet secretory cells (nasal mucosa), type-2 pneumocytes (lungs), and absorptive enterocytes (small intestine), characterizing potential initial target sites for SARS-CoV-2 replication in humans [20]. Taken together SARS-CoV-2 infection can damage the testes and reduce testosterone levels, but the underlying mechanisms are unknown and evidence of virus replication in testicular cells is lacking.

All above discussed facts support that men are more vulnerable to COVID-19 than women. Considering the high chances of sexual transfer of SARS-CoV-2 [21], we planned and conducted an RT-PCR test in the semen of the study participants. Globally, RT-PCR is considered the gold standard test for investigating SARS-CoV-2 in the secretions of COVID-19 patients [4]. All RT-PCR tests done during the first and second semen sampling were negative for SARS-CoV-2 despite the persistence of SARS-CoV-2 in the upper respiratory tract. It was confirmed by the studies done by Rawlings et al. [22] and Pavone et al. [23].

In our study, the semen parameters, such as sperm vitality and total motility, were decreased below the normal limits during the first sampling. In contrast, values of semen agglutination, sperm DFI, semen viscosity, and semen leukocytes were above their normal limits during the second sampling. Other semen parameters like semen volume, sperm concentration, total sperm count, % normal sperm morphology, and semen fructose levels were decreased during the first semen sampling compared to the second semen sampling but within normal limits. In contrast, semen liquefaction time was increased during the first semen sampling compared to the second semen sampling but within the normal limit. The same is confirmed by the study done by Best et al. where SARS-CoV-2 was not found in the semen, but total sperm number (TSN) was decreased during COVID-19 disease [24].

This confirms that COVID-19 disease affects the male reproductive system making semen quality poor. We further noticed that the semen quality assessed after 74 days was improved, but it was of yet poor quality. After 74 days of post-COVID-19, the most affected parameters are semen viscosity, agglutination, vitality, and DFI %. Sperm vitality reflects the proportion of live, membrane-intact spermatozoa determined by Eosin-Nigrosin supravital staining. The reason for a decrease in vitality is the development of reactive oxidative stress (ROS). The male reproductive system balances ROS production and antioxidant activity during a healthy state. During stressful diseases like COVID-19, the overproduction of ROS affect sperm and seminal plasma [25]. Oxidative stress disrupts sperm chromatin leading to DNA damage and an increase in DFI%.

Entry of any pathogen in the body increases levels of leukocytes in the body including seminal plasma rendering the sperms agglutinated [26]. It is advised that all young males suffering from COVID-19 should undergo semen analysis during their post-COVID-19 life. They should undergo subsequent semen analysis after every 74 days till their semen quality returns to normal. Therefore, the question arises, despite SARS-CoV-2 being not shed in the semen of COVID-19 patients then how is it affecting spermatogenesis? One of the primary symptoms of COVID-19 is a high-grade fever, which may disrupt the blood-testis barrier exposing the sperm cells and testicular tissues to circulating cytokines and other inflammatory mediators generated in the body. This may result in a systemic inflammatory state and immune response against the seminiferous epithelium and accessory glands resulting in low semen quality. There was interstitial orchitis with mononuclear inflammatory cells, interstitial edema, and disarrangement of Leydig cells in the testicular autopsies of males who died because of COVID-19 [27].

The researchers also found oligozoospermia, leukospermia, and increased levels of interleukin-6 in some of the patients who had recovered from COVID-19. Increased apoptotic cells and high cytokine levels suggest that the current manifestation occurs in the background of autoimmune orchitis [28]. Recent studies showed positive correlations between ACE2 activity and apoptotic variables with pro-and anti-inflammatory cytokines and ROS. Yang et al. reported the pathological changes in the testis of 12 COVID-19 diseased [29]. The laboratory tests of these cases detected antibody deposition in testicular tissue, but the viral genome of SARS-CoV-2 could not be extracted from the tissue samples. This implies that orchitis induced by SARS-CoV-2 was probably due to an immunological response rather than the direct effect of the virus [6]. Overproduction of inflammatory cytokines caused by viral infections can lead to autoimmune reactions and infiltration of leucocytes, disrupting spermatogenesis and interfering with sex-related hormone secretion [30]. During viral infections, antigens expressed on spermatozoa are foreign to the immune system. Anti-sperm antibodies (ASA) can be formed as a result of different etiologies such as the breakdown of the blood-testis barrier, orchitis, and genital tract inflammation [31]. Possible mechanisms of SARS-CoV-2 infection-induced sperm DNA damage, abnormal motility, systemic oxidative stress, inflammation, and male infertility may occur through disruption of ACE2/Ang (1-7)/Mas and phosphatidylinositol-3-kinase (PI3K)-Akt signaling pathway. Oxidative stress resulting from the overproduction of ROS can profoundly affect the sperm plasma membrane and the subsequent functional integrity of the sperm. Elevated ROS levels are cytotoxic, resulting in a loss of sperm motility, and vitality, increased sperm agglutination, and sperm DNA damage. A recently done study by Salonia et al. suggested that SARS-CoV-2 infection can result in decreased circulating testosterone levels in men with SARS-CoV-2 disease [32].

Overall, the pathophysiology and the long-term impact of SARS-CoV-2 on male fertility are still unclear. Therefore, we suggest a follow-up study further to reveal how long the effect of SARS-CoV-2 on semen quality will persist. A normal result should have a pH level between 7.2 and 7.8. Anything above could indicate an infection and a result less than the normal level could indicate that the sample has been contaminated. PH > 8 was found associated with leukocytospermia [33].

Earlier studies reported that sperm with abnormal morphology may have poor or lack of motility and hence utilizes lower fructose [13]. The low levels of fructose in semen disturb coagulation and sperm movement which could be due to genital tract inflammation [34].

Limitations of the study

A small number of participants, absence of a control group in the research, and the absence of hormonal assessment of the study participants. Our data do not allow a clear interpretation of ACE2 and TMPRSS2 levels as predictors of disease severity. Prospective studies should then be carried out to better explore the prediction effect of these factors on COVID-19 severity. We encourage future studies with greater sample sizes to do so to better define the contribution of ACE2 and TMPRRS2 expression levels on male reproductive outcome severity.

## Conclusions

SARS-CoV-2 is not secreted in the semen during COVID-19 and post-COVID-19 periods and hence does not possess the risk of sexual transmission. Despite the absence of SARS-CoV-2 in the semen, it affected spermatogenesis making semen quality poor. This study suggests that assisted reproductive technology (ART) clinics and sperm banking facilities should consider assessing detailed semen analysis of males with a history of COVID-19.

## References

[1] Wu YC, Chen CS, Chan YJ (2020). The outbreak of COVID-19: an overview. J Chin Med Assoc.

[2] Meselson M (2020). Droplets and aerosols in the transmission of SARS-CoV-2. N Engl J Med.

[3] Guan WJ, Ni ZY, Hu Y (2020). Clinical characteristics of coronavirus disease 2019 in China. N Engl J Med.

[4] Kevadiya BD, Machhi J, Herskovitz J (2021). Diagnostics for SARS-CoV-2 infections. Nat Mater.

[5] Purpura LJ, Alukal J, Chong AM (2022). SARS-CoV-2 RNA shedding in semen and oligozoospermia of patient with severe coronavirus disease 11 weeks after infection. Emerg Infect Dis.

[6] Xu J, Qi L, Chi X (2006). Orchitis: a complication of severe acute respiratory syndrome (SARS). Biol Reprod.

[7] Sigurdardóttir OG, Kolbjørnsen O, Lutz H (2001). Orchitis in a cat associated with coronavirus infection. J Comp Pathol.

[8] Fan C, Lu W, Li K, Ding Y, Wang J (2020). ACE2 expression in kidney and testis may cause kidney and testis infection in COVID-19 patients. Front Med (Lausanne).

[9] Franken DR, Oehninger S (2012). Semen analysis and sperm function testing. Asian J Androl.

[10] Lai CK, Lam W (2021). Laboratory testing for the diagnosis of COVID-19. Biochem Biophys Res Commun.

[11] Boivin J, Takefman J, Braverman A (2011). The fertility quality of life (FertiQoL) tool: development and general psychometric properties. Hum Reprod.

[12] Cooper TG, Noonan E, von Eckardstein S (2010). World Health Organization reference values for human semen characteristics. Hum Reprod Update.

[13] Agarwal A, Gupta S, Sharma R (2016). Qualitative Seminal Fructose. Andrological Evaluation of Male Infertility.

[14] Toragall MM, Satapathy SK, Kadadevaru GG, Hiremath MB (2019). Evaluation of seminal fructose and citric acid levels in men with fertility problem. J Hum Reprod Sci.

[15] Riedel HH (1980). Techniques for the detection of leukocytospermia in human semen. Arch Androl.

[16] Agarwal A, Gupta S, Sharma R (2016). Eosin-Nigrosin Staining Procedure. Andrological Evaluation of Male Infertility.

[17] Björndahl L, Söderlund I, Johansson S, Mohammadieh M, Pourian MR, Kvist U (2004). Why the WHO recommendations for eosin-nigrosin staining techniques for human sperm vitality assessment must change. J Androl.

[18] Beyerstedt S, Casaro EB, Rangel ÉB (2021). COVID-19: angiotensin-converting enzyme 2 (ACE2) expression and tissue susceptibility to SARS-CoV-2 infection. Eur J Clin Microbiol Infect Dis.

[19] Chen Y, Guo Y, Pan Y, Zhao ZJ (2020). Structure analysis of the receptor binding of 2019-nCoV. Biochem Biophys Res Commun.

[20] Moshrefi M, Ghasemi-Esmailabad S, Ali J, Findikli N, Mangoli E, Khalili MA (2021). The probable destructive mechanisms behind COVID-19 on male reproduction system and fertility. J Assist Reprod Genet.

[21] Huang C, Wang Y, Li X (2020). Clinical features of patients infected with 2019 novel coronavirus in Wuhan, China. Lancet.

[22] Gonzalez DC, Khodamoradi K, Pai R (2020). A systematic review on the investigation of SARS-CoV-2 in semen. Res Rep Urol.

[23] Pavone C, Giammanco GM, Cascino AP (2022). Assessment of SARS-CoV-2 RNA shedding in semen of 36 males with symptomatic, asymptomatic, and convalescent infection during the first and second wave of COVID-19 pandemic in Italy. Asian J Androl.

[24] Best JC, Kuchakulla M, Khodamoradi K (2021). Evaluation of SARS-CoV-2 in human semen and effect on total sperm number: a prospective observational study. World J Mens Health.

[25] Sengupta P, Dutta S (2020). Does SARS-CoV-2 infection cause sperm DNA fragmentation? Possible link with oxidative stress. Eur J Contracept Reprod Health Care.

[26] Aitken RJ, Baker HW (1995). Seminal leukocytes: passengers, terrorists or good samaritans?. Hum Reprod.

[27] Gupta R, Kumar M, Dhayal IR (2022). COVID-19 and genitourinary tract: a retrospective study in the tertiary care center. Cureus.

[28] Li H, Xiao X, Zhang J (2020). Impaired spermatogenesis in COVID-19 patients. EClinicalMedicine.

[29] Hajizadeh Maleki B, Tartibian B (2021). COVID-19 and male reproductive function: a prospective, longitudinal cohort study. Reproduction.

[30] Hedger MP, Meinhardt A (2003). Cytokines and the immune-testicular axis. J Reproduct Immunol.

[31] Archana SS, Selvaraju S, Binsila BK, Arangasamy A, Krawetz SA (2019). Immune regulatory molecules as modifiers of semen and fertility: a review. Mol Reprod Dev.

[32] Salonia A, Pontillo M, Capogrosso P (2022). Testosterone in males with COVID-19: a 7-month cohort study. Andrology.

[33] Kulkarni S, Kulkarni NV (2015). Study of semen parameters in male partners among infertile couples. Int J Reproduct Contracept Obstetr Gynecol.

[34] Henkel RR (2011). Leukocytes and oxidative stress: dilemma for sperm function and male fertility. Asian J Androl.

